# The clinical effectiveness of staple line reinforcement with different matrix used in surgery

**DOI:** 10.3389/fbioe.2023.1178619

**Published:** 2023-06-07

**Authors:** Wei Jing, Yiqian Huang, Jin Feng, Hongyang Li, Xueqiao Yu, Bo Zhao, Pengfei Wei

**Affiliations:** Beijing Biosis Healing Biological Technology Co., Ltd., Beijing, China

**Keywords:** staple line reinforcement, bovine pericardium, small intestinal submucosa (SIS), polyglycolic acid (PGA), expanded polytetrafluoroethylene (ePTFE)

## Abstract

Staplers are widely used in clinics; however, complications such as bleeding and leakage remain a challenge for surgeons. To tackle this issue, buttress materials are recommended to reinforce the staple line. This Review provides a systematic summary of the characteristics and applications of the buttress materials. First, the physical and chemical properties of synthetic polymer materials and extracellular matrix used for the buttress materials are introduced, as well as their pros and cons in clinical applications. Second, we review the clinical effects of reinforcement mesh in pneumonectomy, sleeve gastrectomy, pancreatectomy, and colorectal resection. Based on the analysis of numerous research data, we believe that buttress materials play a crucial role in increasing staple line strength and reducing the probability of complications, such as bleeding and leakage. However, considering the requirements of bioactivity, degradability, and biosafety, non-crosslinked small intestinal submucosa (SIS) matrix material is the preferred candidate. It has high research and application value, but further studies are required to confirm this. The aim of this Review is to provide comprehensive guidance on the selection of materials for staple line reinforcement.

## Highlights

• Most studies have concluded that using a reinforcement patch for the staple line is effective in strengthening it and reducing the incidence of postoperative complications, such as bleeding and staple line leakage.
• A staple line reinforcement patch can be prepared using non-degradable synthetic polymer expanded polytetrafluoroethylene (ePTFE), degradable synthetic polymer polyglycolic acid (PGA) and its copolymers, crosslinked bovine pericardium (BP) extracellular matrix, and non-crosslinked small intestinal submucosa (SIS) extracellular matrix. The various physical and chemical properties of these materials result in different levels of biocompatibility for the meshes.
• The non-degradability of ePTFE and crosslinked BP staple line reinforcement patches often leads to more postoperative complications in clinical practice, while PGA patches are prone to cause inflammation after degradation.
• Compared with the aforementioned three materials, the SIS staple line reinforcement patch exhibits superior biocompatibility and biosafety.

## Introduction

The introduction of staplers has significantly advanced the fields of general surgery and minimally invasive surgery. These medical devices use metal staples to cut and anastomose tissues. Currently, staplers are widely used for clinical operations on the lungs, stomach, intestine, and pancreas as they improve operational efficiency and shorten surgery time, and are recommended for surgeons to use (Baker et al., 2004; Head and McKay, 2018). The rigid nature of metal staplers can sometimes result in incomplete tissue anastomosis, which might be attributed to the existence of stress concentration between the rigid staplers and soft fragile tissues, leading to staple failure (Wang et al., 2022). As a consequence, complications such as bleeding, leakage, stenosis, scarring, and secondary infections may arise, necessitating additional surgeries that can impose significant financial and psychological burdens on patients and pose a threat to their health and wellbeing (Choi et al., 2012; Chekan and Whelan, 2014).

In recent years, there have been considerable improvements in staplers, but the benefits of staple line reinforcement as the latest surgical anastomosis technology cannot be ignored. Staple line reinforcement patches are used with a stapler for surgical operations. After activating the stapler, two rows of staples penetrate the reinforcement material and interlock with the tissue, securely sewing the reinforcement patch to the tissue. The hemostatic effect of the reinforcement materials mainly comes from their compression effect on the cross-cut tissue, which can improve vascular sealing (Nguyen et al., 2005). The main potential advantage of reinforcement patches is to improve the strength of the staple line, enhance vascular sealing, and minimize the risk of anastomotic leakage (D'Ugo et al., 2014).

**FIGURE 1:**
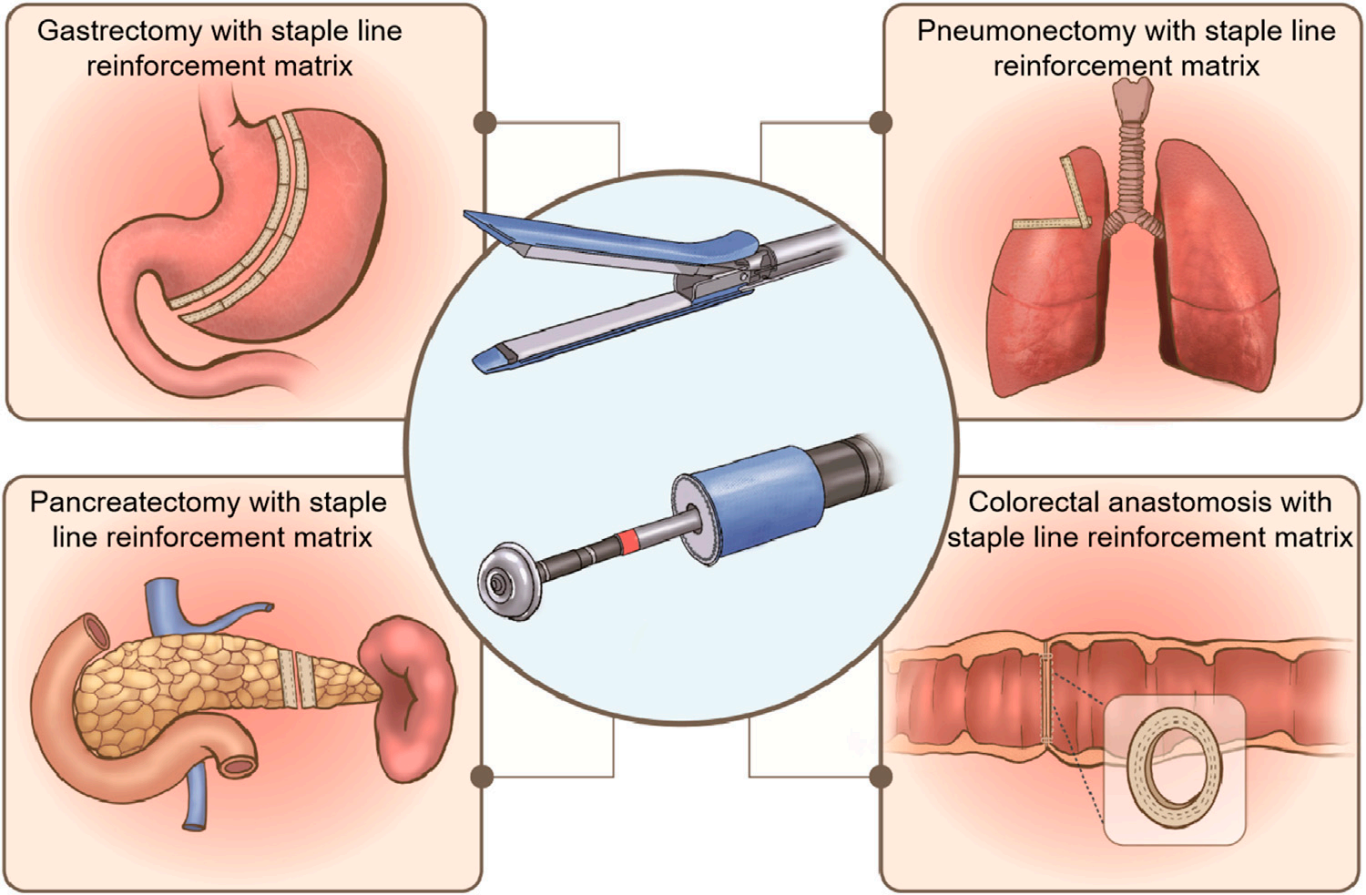
Staple line reinforcement patches used for gastrectomy, pneumonectomy, pancreatectomy, and colorectal anastomosis.

Staple line reinforcement buttress materials (Dang et al., 2021), tissue adhesives (Carandina et al., 2016), and oversewing treatments (Rogula et al., 2015) are being used in clinical trials to reduce complications after anastomosis. Among these options, buttress materials have been studied the most. In general, staple line reinforcement materials can be divided into two categories: synthetic polymers and natural extracellular matrix (ECM). Commercial products of staple line reinforcement materials include expanded polytetrafluoroethylene (ePTFE) (Seamguard^®^, W. L. Gore and Associates, United States), synthetic polyglycolic acid (PGA) (Neoveil™, Gunze Ltd., Japan), copolymer PGA and tri-methylene carbonate (PGA/TMC) (Seamguard^®^, W. L. Gore and Associates, United States), as well as ECM, such as bovine pericardium (BP, Peri-strips^®^, Synovis Life Technologies, United States) and small intestinal submucosa (SIS, Biodesign^®^, Cook Medical, United States). These products have been used for staple line reinforcement in various areas, such as the stomach, colorectum, lungs, and pancreas, as illustrated in Figure 1.

The objective of this study was to identify the effect of the various types of materials used for reinforcing the staple line during pulmonary resection, distal pancreatic anastomosis, sleeve gastrectomy, and colorectal anastomosis by reviewing the relevant literature.

## Materials for staple line reinforcement patches

Staple line reinforcement materials have been classified as synthetic polymers and animal-derived ECM materials. Synthetic polymer materials include non-degradable ePTFE and degradable PGA and its copolymers, while ECM materials include BP and SIS. The advantages and disadvantages of these materials are listed in Table 1.

**TABLE 1:** The advantages and disadvantages of different staple line reinforcement materials.

Item	Products	Advantages	Disadvantages
ePTFE	Seamguard^®^	Excellent chemical stability, thermal resistance, and bio-inertia	Not degradable
PGA and its copolymers	Seamguard^®^, Neoveil™	Biocompatibility and degradability	Can cause inflammation
BP	Peri-strips^®^	Low immunogenicity and good mechanical properties	Potential cytotoxicity and difficult to degrade or cannot degrade
SIS	Biodesign^®^	Bioactivity and degradability	

### Synthetic polymer materials

#### ePTFE

ePTFE is derived from PTFE through a specialized molding process and consists of carbon and fluorine elements. The repeating unit is -CF_2_-, which imparts remarkable chemical resistance, thermal stability, hydrophobicity, and bio-inertness. ePTFE is biocompatible and has been extensively used in various biomedical fields, such as in vascular implants (Yamamoto et al., 2020), vocal cord repair (Bernal-Sprekelsen et al., 2004), bone repair (Fang et al., 2003), and heart repair (Hodonsky et al., 2015). However, ePTFE cannot be degraded; if it remains implanted *in vivo* for an extended period, it may elicit a foreign body reaction (Wood et al., 2013).

#### PGA

PGA is a linear polyhydroxy acid ester with carboxyl and hydroxyl groups. It can be easily hydrolyzed and degraded into metabolic intermediates, such as lactic acid and glycolic acid (Zhang et al., 2020a). Lactic acid can be metabolized into carbon dioxide and water *in vivo*, while glycolic acid can be excreted via urine after participating in the tricarboxylic acid cycle. PGA is recognized as a safe and biodegradable material in Europe, America, and Japan (Maurus and Kaeding, 2004) and is widely used in absorbable sutures (Munteanu et al., 2017), tissue repair materials (Tariverdian et al., 2019), drug-release carriers (Lee et al., 2010) etc. However, PGA has some clinical issues, such as rapid degradation, which may cause inflammation and severe postoperative tissue adhesion (Lin et al., 2017; Zhang et al., 2020b). Thus, there are certain safety concerns associated with the clinical use of PGA.

#### Animal-derived ECM

With the increasing use of tissue engineering technology, scaffolding materials have become an important focus of research. These materials are required to provide mechanical support and the necessary microenvironment for cell growth, migration, and differentiation. As a natural scaffolding material, ECM has excellent biocompatibility, low immunogenicity, and a low risk of rejection and infection, making it a promising material for tissue engineering research and clinical applications (Liu et al., 2022). ECM provides physical support and a suitable microenvironment for cell growth and can modulate cell adhesion, proliferation, and differentiation through signal transduction (Bandzerewicz and Gadomska-Gajadhur, 2022). Fabrication of ECM scaffolds can be achieved by removing host cells and preserving the residual ECM morphology through various methods. The main components of ECM show similar structures to the natural structure of ECM and have excellent biocompatibility and bioactivity. Currently, ECM from the dermis, bladder, BP, and SIS have been used in clinical applications, with the staple line reinforcement patches of SIS and BP having been used clinically for many years.

#### BP

BP is a unique biomaterial with distinct biochemical properties. It primarily consists of collagen fibers and elastic structures, and its internal connective tissue contains a substantial amount of structural protein. Consequently, it provides the required mechanical support for cell migration and revascularization during the repair process (Grebenik et al., 2020). BP has extensive clinical applications. To enhance the mechanical properties and reduce the immunogenicity of BP materials, chemical crosslinking strategies have been extensively employed in BP material construction. Glutaraldehyde is the most commonly used crosslinking agent for BP materials due to its low cost, high water solubility, and reactivity (Calero et al., 2002). Presently, in the regenerative medicine field, glutaraldehyde-crosslinked BP products have been applied in cardiovascular (Prabhu et al., 2017), urology (Moon et al., 2011), ophthalmology (Bajaj and Pushker, 2002) etc. More than 70% of BP material is composed of collagen, enabling glutaraldehyde to react with the lysine amino group in collagen, forming a robust covalent bond, thus reducing the immunogenicity of BP while enhancing its mechanical properties (Griffiths et al., 2008). However, the use of glutaraldehyde for chemical crosslinking also has its drawbacks, including the cytotoxicity of its chemical agents (Oswal et al., 2007), its non-degradability or reduced degradability (van den Heever et al., 2013), and susceptibility to calcification *in vivo* (Ohri et al., 2004).

#### SIS

SIS, a decellularized porcine small intestinal submucosa, is an ECM material that contains various bioactive factors, such as vascular endothelial growth factor (VEGF), fibroblast growth factor (FGF), and epidermal growth factor (EGF) (Wang et al., 2016). These factors can promote tissue repair and regeneration (Lin et al., 2015). Additionally, SIS has a fibrous network scaffold structure composed of collagen fibers, which promotes cell adhesion and proliferation (Ji et al., 2019). As a bioactive scaffold, SIS has superior bioactivity and biocompatibility compared with synthetic materials, and can induce angiogenesis and wound regeneration (Hodde et al., 2001; Gilbert et al., 2006). It has been demonstrated that SIS effectively repairs damaged tissues and can be metabolized and completely replaced by regenerated tissue (Badylak et al., 2002), reducing the risk of discomfort, immune rejection, migration, or erosion associated with permanent materials. SIS has been widely used in the biomedical field for decades, particularly in the treatment of abdominal wall hernias (Ma et al., 2015), vascular and ureteral reconstruction (Smith et al., 2002; Feliciano, 2004), vocal cord repair (Choi et al., 2014) and other fields, with increasing demand.

## Staple line reinforcement patches used for surgery

### Pneumonectomy

During laparoscopic pulmonary resection, the use of staples often results in small holes in the lung parenchyma and subsequent air leakage (Murray et al., 2002). This may also occur when the lung is pulled and torn during re-expansion. Prolonged air leakage (PAL) is a common complication following pneumonectomy, with an incidence rate of approximately 15% (Downey et al., 2006). Several studies have demonstrated that PAL can increase the duration of chest tube use (Vannucci et al., 2016), length of hospital stay (Safranek et al., 2005), medical costs (Nishida et al., 2017), and risk of infectious complications, such as pneumothorax and pneumonia (Murakami et al., 2018). To address this issue, various staple line reinforcement patches have been developed, and a comparative analysis of these products is presented in Table 2.

**TABLE 2:** Experiments of different staple line reinforcement patches for pneumonectomy.

Researcher	Group	Model	Result
Cecil C. Vaughn et al. (Vaughn et al., 1998)	ePTFE, BP	Lung volume reduction surgery in canines	No air leakage, inflammation, and less tissue coverage in BP. No inflammation and more tissue coverage in ePTFE
Kevin D. Murray et al. (Murray et al., 2002)	ePTFE, BP, control	Lung volume reduction surgery in human cadavers	Compressive strength of reinforced lung tissue: control < BP < ePTFE
Douglas et al. (Downey et al., 2006)	ePTFE, PGA/TMC, BP, SIS, control	Lung resections in swine models	Only SIS could significantly improve the compressive strength compared with the control
Jacopo Vannucci et al. (Vannucci et al., 2016)	BP + collagen, control	Pneumonectomy in the swine model	Strength of staple line: BP + collagen < control, BP caused serious inflammation at the staple line, prevented wound healing

In early animal experiments, a comparative analysis was conducted on canine pneumonectomy using ePTFE and BP (Vaughn et al., 1998). The results showed that at 30 days postoperatively, there was focal chronic inflammation and less new tissue coverage at the BP implantation site, while there was no focal inflammation at the ePTFE implantation site, with thicker new tissue coverage. At 95 and 167 days postoperatively, the inflammation gradually declined in the BP groups, but the amount of tissue coverage remained low and BP was not absorbed by the surrounding tissues, while the thickness of newly formed tissues significantly increased in the ePTFE groups. In addition, research has shown that BP may prolong the healing process of broken ends of the staple line and cause an obvious inflammation response at 60 days postoperatively, with large amounts of granulation tissues presented in the operative locations (Vannucci et al., 2016). Kevin D. Murray et al. reinforced human cadaveric lung tissue using ePTFE and BP and then tested the pressure resistance (Murray et al., 2002). Their study showed that some unreinforced staple lines tended to leak air at a pressure of 20 mm Hg, and more than 90% of the samples failed at a pressure of 35 mm Hg. Both BP and ePTFE could reduce the possibilities of air leakage at 35 mm Hg compared with the unreinforced ones, and ePTFE was superior to BP at a pressures of 40 mm Hg. D. M. Downey et al compared the lung sealing performances of ePTFE, PGA/TMC, BP, and SIS staple line reinforcement patches (Downey et al., 2006). The results showed that staple lines reinforced with SIS had the highest leaking pressure of 75 cm H_2_O and were significantly better than those with the three other materials and the unreinforced group.

Some studies have investigated the clinical use of PGA and BP materials in lung resection anastomosis. For example, Lim E. et al. examined adverse events associated with medical devices in external thoracic surgery for lung cancer and found minimal safety concerns with the use of the PGA staple line reinforcement patch compared with the non-reinforced group (Lim et al., 2022). Although many animal experiments have demonstrated that BP can improve the strength of the staple line, adverse events related to BP materials have also been reported. In one case, a patient coughed up blood clots with patch fragments and staples 3 months after pulmonary resection (Shamji et al., 2002), and in other case, the migration of an intact patch caused an observation pneumonia and recurrent hemoptysis over several years (Provencher, 2003).

The published articles indicate that BP buttress materials have the potential to improve the strength of the staple line in lung resection, but it is important to consider postoperative complications, such as inflammation response and rejection reactions.

### Pancreatectomy

Pancreatic fistula is a major complication after pancreatic resection, and its incidence can be high, ranging from 16% to 36% after distal pancreatectomy. However, the use of absorbable reinforcement patches for distal pancreatic anastomosis has been shown to significantly reduce the incidence of postoperative pancreatic fistula (POPF), with some studies reporting no cases of POPF (Table 3). (Jimenez et al., 2007; Thaker et al., 2007; Guzman et al., 2009; Johnston et al., 2009; Yamamoto et al., 2009; Hamilton et al., 2012; Wennerblom et al., 2021) Meta-analysis studies have also confirmed that reinforcement patches reduce the incidence of POPF (Jensen et al., 2013), mortality, postoperative bleeding, and the need for secondary surgery (Oweira et al., 2022). Although some studies have suggested it is unclear whether staple line reinforcement after distal pancreatectomy can prevent biochemical pancreatic fistula, it has been shown to significantly reduce the incidence of clinically relevant POPF compared with standard stapling without reinforcement (Elkomos et al., 2022).

**TABLE 3:** Staple line reinforcement patches used for distal pancreatectomy.

Researcher	Group	Incidence of POPF
Thaker, RI, et al. (Thaker et al., 2007)	Absorbable mesh, control	3.5% (29) in which mesh reinforcement was utilized, and 36% (11) in the control (*p* < 0.005)
Jimenez, RE, et al. (Jimenez et al., 2007)	Seamguard^®^, control	0% (13) in which mesh reinforcement was utilized, and 39% (18) in the control (*p* = 0.025)
Guzman, EA, et al. (Guzman et al., 2009)	Seamguard^®^, control	73% (15) in which mesh reinforcement was utilized, and 20% (15) in the control (*p* = 0.005)
Mc Johnston, et al. (Johnston et al., 2009)	Absorbable mesh, control	10% (70) in which mesh reinforcement was utilized, and 25% (99) in the control (*p* < 0.02)
Hamilton, NA, et al. (Hamilton et al., 2012)	Seamguard^®^, Peristrips Dry^®^, control	1.9% (53) in which mesh reinforcement was utilized, and 20% (45) in the control (*p* = 0.0007)
Yamamoto, M, et al. (Yamamoto et al., 2009)	Seamguard^®^, control	4% (38) in which mesh reinforcement was utilized, and 26% (47) in the control (*p* = 0.01)
Wennerblom, J, et al. (Wennerblom et al., 2021)	Seamguard^®^, control	11% (56) in which mesh reinforcement was utilized, and 16% (50) in the control (*p* = 0.332)

### Gastrectomy

With the rising prevalence of morbid obesity, laparoscopic sleeve gastrectomy (LSG) has emerged as a crucial weight loss procedure. During this surgery, the greater curvature of the stomach is vertically excised using laparoscopy, creating a smaller gastric sac. This restriction of the stomach volume helps patients feel full, while the removal of ghrelin-producing cells located at the bottom of the stomach reduces their appetite. However, staple line-related complications, including bleeding and staple line leakage, pose significant challenges for bariatric surgeons. To mitigate these complications, staple line reinforcement patches made of PGA copolymer (Noel et al., 2016), BP (Stamou et al., 2011), and SIS are widely used in LSG. Several clinical investigations have reported the efficacy of PGA patches in reducing intraoperative bleeding and shortening hospital stays (Iannelli et al., 2022), while BP patches have shown similar results in these aspects (Stamou et al., 2011; Moon et al., 2013). The clinical practice guidelines for bariatric surgery published by the European Society for Endoscopic Surgery (EAES) in 2020 recommended the use of an anastomosis reinforcement patch during sleeve gastrectomy to minimize perioperative complications, including overall mortality and bleeding incidence (Di Lorenzo et al., 2020).

Researchers have conducted studies to compare the efficacy of various staple line reinforcement patches. A meta-analysis by Scott A. Shikora et al. showed that cases without reinforcement products had high bleeding (3.45%) and staple line leakage rates (2.72%), whereas the rates were significantly lower in cases with reinforcement products (Table 4) (Shikora and Mahoney, 2015). Among these products, the use of BP patches resulted in the lowest rates of bleeding and staple line leakage, indicating BP patches were superior to suture or synthetic staple line reinforcement products. Morris J. Washington et al. (Washington et al., 2019) conducted a retrospective study on 722 cases to investigate the bleeding and staple line leakage rates after LSG with SIS staple line reinforcement patches. The results showed that SIS staple line reinforcement patches reduced the bleeding rate to 1.2% and the staple line leakage rate to 0.6%. These large-scale data analyses suggested that BP and SIS could effectively reduce the incidence of staple line bleeding and are superior to synthetic polymer materials.

**TABLE 4:** Incidence of staple line complications in LSG (Shikora and Mahoney, 2015; Washington et al., 2019).

	Leakage		Bleeding rate	
	Incidence	No. patients	Incidence	No. patients
None	3.27%	3,958	4.94%	2,865
Over-suturing	2.70%	6,141	2.41%	4,682
Glycolide copolymer	3.25%	1,850	2.09%	1,997
BP	1.83%	1,678	1.16%	1,632
SIS	0.6%	722	1.2%	722

Both BP and SIS are ECM derived from animal tissue, and collagen is their main component. Collagen has been widely studied for its hemostatic properties and is frequently used in clinical therapy (Liu et al., 2020), affording BP and SIS an advantage in terms of hemostasis. However, most BP patches are chemically crosslinked, which can result in residual crosslinking agent stimulating an inflammatory response in lung and stomach tissues, leading corrosion and diffusion migration after laparoscopic gastric bypass surgery (Portillo and Franklin, 2010), as well as other complications in the late postoperative period. One case report even described the fragments of BP patches being found in a patient’s vomit 4 weeks after LSG (Consten et al., 2004). Additionally, a retrospective study found that although the use of BP patches in LSG reduces bleeding and shortens hospital stays, it increases the risk of postoperative anastomotic leakage and is associated with statistically higher rates of patient readmission and reoperation (Barreto et al., 2015).

### Colorectal anastomosis

Anastomosis leakage or rupture after colorectal surgery can have serious consequences, such as local tumor recurrence and anastomotic stenosis. The use of staple line reinforcement patches has been shown to reduce the incidence of small bowel obstruction and anastomotic stenosis (Shikora and Mahoney, 2015). The rupture strength of the colorectal staple line reinforced with BP material is higher than that of the adjacent intestinal tissue (Washington et al., 2019). Animal studies have demonstrated the safety and efficacy of PGA in colorectal anastomosis (Kimura et al., 2021), but some researchers have reported that PGA reinforcement patches may cause peritonitis in the colorectal staple line (Henne-Bruns et al., 1990). *In vitro* experiments have shown that the bursting pressure of the reinforced intestinal staple line is approximately 200 mm Hg without the use of reinforcement material, whereas BP buttress material can increase the burst pressure to 362 mm Hg and SIS buttress material can increase the burst pressure to 441 mm Hg (Table 5) (Pinheiro et al., 2006; Gaertner et al., 2010). Furthermore, animal experiments have shown that SIS material can promote colonic wound healing and does not cause abdominal adhesion and other complications (Hoeppner et al., 2009).

**TABLE 5:** Comparison of SIS and BP materials (Pinheiro et al., 2006; Gaertner et al., 2010).

	Animal	Tissue	Crosslink	Mean burst pressure
SIS	Pig	Small intestine submucosal tissue	Non-crosslinking	EG: 441.33 mm Hg
				CG: 209.26 mm Hg
BP	Bovine	Pericardium	Chemical crosslinking	EG: 362 mm Hg
				CG: 204 mm Hg

EG, group with buttress material reinforcing; CG, group without reinforcing.

Animal-derived staple line reinforcement patches are made from decellularized ECM of animal tissue. Among them, SIS material has a uniform thickness and combines the flatness of synthetic polymer membrane with high biological activity. On the other hand, BP material is derived from bovine pericardium, which contains a significant amount of fat and fibrous structures, leading to uneven material thickness (Stieglmeier et al., 2021). This uneven thickness may result in uneven stress on the staple line, resulting in a lower strength than SIS material. Therefore, SIS material is more attractive than BP material in clinical applications as a new generation decellularized biomaterial with better performance for staple line reinforcement.

## Conclusion

Most studies suggest that staple line reinforcement materials can reduce the incidence of staple line leakage and bleeding. While some doubts remain, it is certain that staple line reinforcement patches can effectively improve the strength of the staple line and avoid staple line leakage caused by common clinical factors.

The clinical studies discussed in this Review have shown that staple line reinforcement patches are effective in reducing the incidence of postoperative complications in various procedures, such as pneumonectomy, pancreatectomy, gastrectomy, and colorectal anastomosis. Additionally, some studies have shown that staple line reinforcement patches made of different materials may behave differently in terms of tissue regeneration and inflammatory stimulation, which is related to the chemical properties of the materials. In terms of synthetic materials, although ePTFE has good bio-inertia and biocompatibility, its non-degradability may lead to foreign body reactions. PGA, on the other hand, is a polymer material with excellent biodegradability and has been used in various clinical fields for many years. In terms of ECM materials, BP materials have good mechanical properties, but the use of a toxic crosslinking agent can result in cytotoxicity and non-degradability. Comparatively, SIS materials exhibit excellent bioactivity, biocompatibility, and biodegradability. In this Review, we did not focus on the mechanical properties of staple line reinforcement patches because the mechanical properties of currently applied materials can generally meet the demands of staple line reinforcement. Moreover, there is no systematic comparative study on the mechanical properties of staple line reinforcement patches made of various materials nor any study on the effect of reinforcement of materials with different mechanical properties. The influence of the mechanical strength of staple line reinforcement patches on the reinforcement and repair effect requires further investigation.

In the development and clinical application of staple line reinforcement patches, safety and degradability are the crucial factors, regardless of whether the material is synthetic or ECM based. As a result, PGA and its copolymers, as well as SIS, are the preferred options for staple line reinforcement patches. However, as PGA materials can cause inflammation in the body, non-crosslinked decellularized SIS material appears to be the optimal choice for staple line reinforcement patches. At present, there is limited research on the clinical use of SIS materials in staple line reinforcement and further investigation is urgently needed.
